# Supplementary figures and images for: 17β-estradiol upregulates oxytocin and the oxytocin receptor in C2C12 myotubes
Source: PeerJ. 2017 Mar 30;5:e3124. doi: 10.7717/peerj.3124 (PMC5376115; doi:10.7717/peerj.3124)

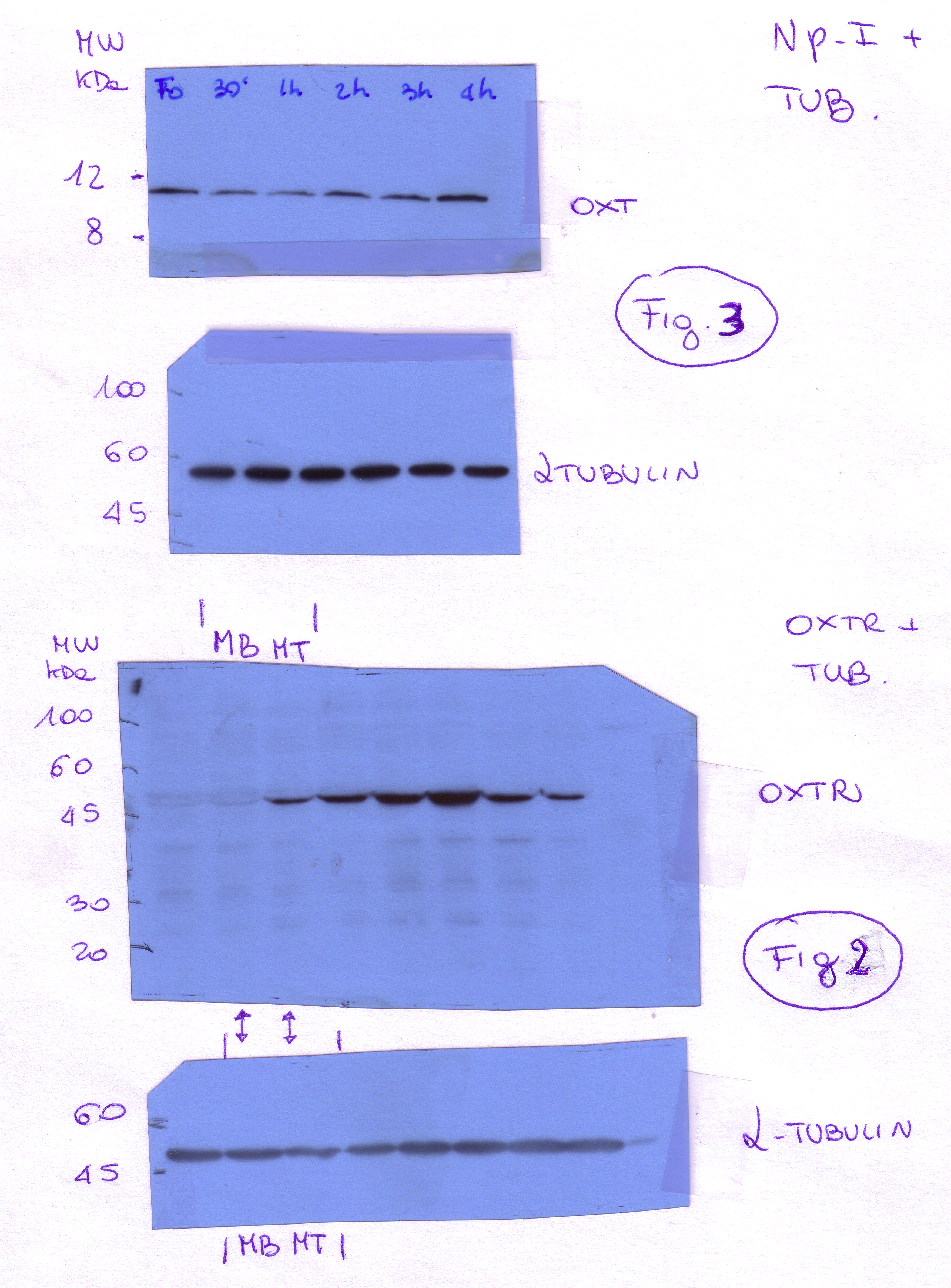

Supplement: Supplemental Information 2 [file peerj-05-3124-s002.tif]

kDa  
20  
12  
8

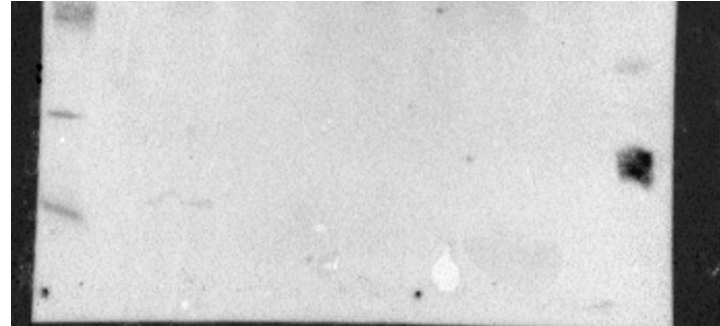

← Np-I

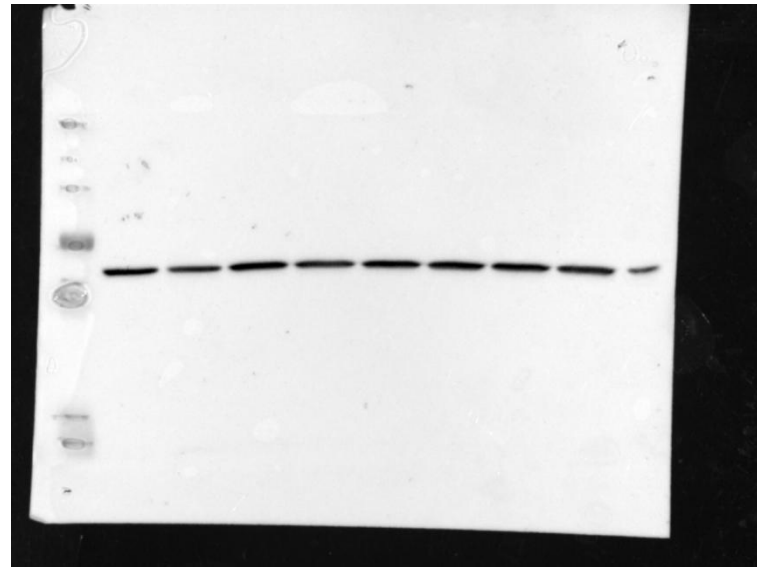

← tub

Supplement: Supplemental Information 3 [file peerj-05-3124-s003.pdf]

OXTR rep 1

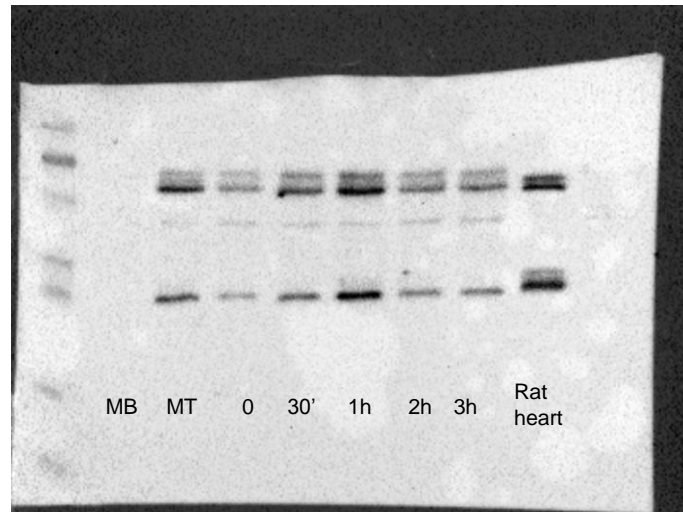

OXTR rep 2

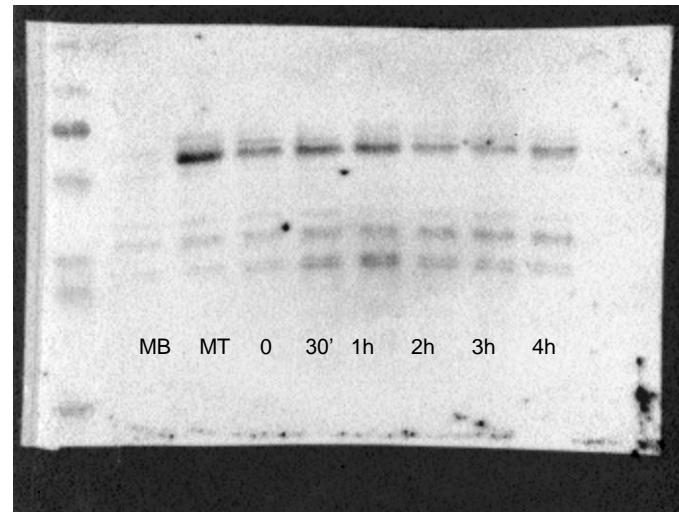

Tubulin rep 1

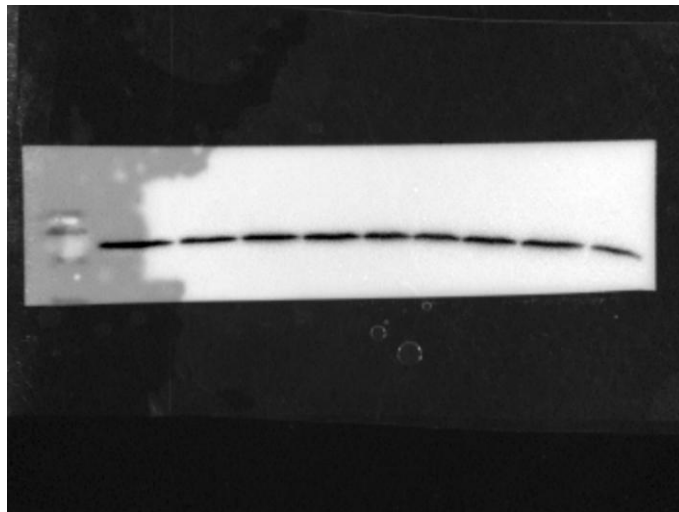

Tubulin rep 2

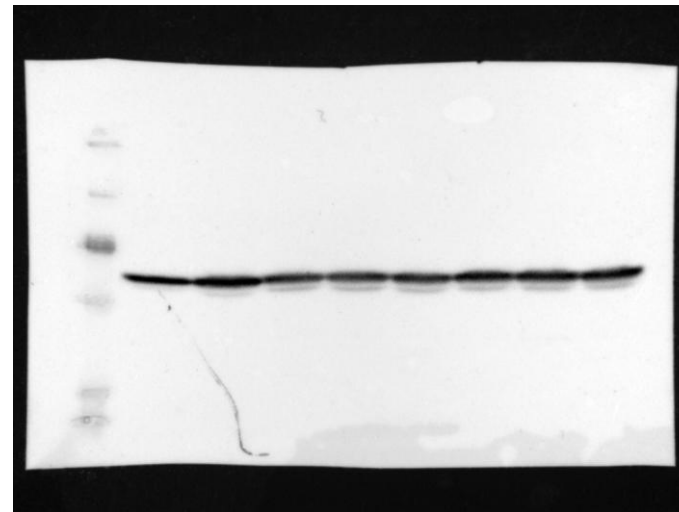

Supplement: Supplemental Information 4 [file peerj-05-3124-s004.pdf]
